# Latent profile and determinants of self-management behaviors among older adult patients with chronic diseases: a cross-sectional study

**DOI:** 10.3389/fpubh.2025.1506545

**Published:** 2025-02-05

**Authors:** Yu Jiao Shao, Xiao Cui Duan, Xue Jun Xu, Hong Yan Guo, Ze Yu Zhang, Shuang Zhao, Fu Zhi Wang, Yong Xia Chen, Qin Chen, Shi Qing Zhang, Xiu Mu Yang

**Affiliations:** ^1^School of Nursing, Bengbu Medical University, Bengbu, Anhui, China; ^2^Department of General Practice, First Affiliated Hospital, Bengbu Medical University, Bengbu, Anhui, China; ^3^Nursing Department, The 902nd Hospital of Joint Logistic Support Force of PLA, Bengbu, Anhui, China; ^4^Nursing Department, First People's Hospital Affiliated with Bengbu Medical University, Bengbu, Anhui, China; ^5^School of Health Management, Bengbu Medical University, Bengbu, Anhui, China; ^6^Nursing Department, First Affiliated Hospital, Bengbu Medical University, Anhui, China; ^7^Nursing Department, Suzhou Hospital Affiliated with Anhui Medical University, Suzhou, Anhui, China

**Keywords:** older adult, chronic diseases, self-management, determinants, latent profile analysis

## Abstract

**Objective:**

To explore latent profiles of self-management behaviors in older adult patients with chronic diseases and identify the factors that influence different profiles, guiding targeted interventions.

**Methods:**

This study used convenience sampling to recruit 536 older adult patients with chronic diseases from three tertiary hospitals in Anhui Province between October 2023 and May 2024. Data were collected using a general information questionnaire, the age-adjusted Charlson Comorbidity Index (aCCI), the Chronic Disease Self-Management Behavior Scale, the Chronic Disease Management Self-Efficacy Scale, the Psychological Status Scale, the Digital Health Literacy Scale, and the Social Support Scale. Latent profile analysis was conducted using Mplus 8.3, and univariate and multivariate logistic regression analyses were performed using SPSS 26.0.

**Results:**

Three profiles of self-management behaviors emerged: “Low Self-Management” (50.2%), “High Exercise and Cognitive Management” (8.6%), and “Moderate Management with Enhanced Communication” (41.2%). Multivariate logistic regression revealed that residence, aCCI, number of digital devices used, perceived usefulness of digital health information, digital health literacy, social support, chronic disease management self-efficacy, and psychological status were significant factors affecting self-management profiles (all *p* < 0.05).

**Conclusion:**

Self-management behaviors in older adult patients with chronic diseases were generally low, with substantial heterogeneity across profiles. Healthcare providers should tailor interventions based on the characteristics of each group to enhance self-management in digital health contexts.

## 1 Introduction

With the accelerating aging of China's population, the older adult demographic continues to expand (1). By the end of 2023, the population aged 60 and above reached 297 million, accounting for 21.1% of the total population, while those aged 65 and above numbered 217 million, representing 15.4% of the total (2). This proportion is projected to rise to 30% by 2050 (3). As the population ages, the prevalence of chronic non-communicable diseases (chronic diseases, NCDs) is also increasing. Due to their high prevalence, long duration, low control rates, and significant economic burden, NCDs have become a major public health issue, threatening individual health and hindering socio-economic development. Globally, NCDs cause ~41 million deaths annually, accounting for 74% of all deaths (4). In China, over 80% of deaths are attributed to NCDs. Older adult individuals with chronic diseases often face high prevalence rates, multiple comorbidities, mental health challenges, and elevated disability and mortality rates, making self-management particularly complex (1).

Chronic Disease Self-Management (CDSM) involves patients, guided by healthcare professionals, adopting self-management strategies to control disease progression. These strategies include preventive and therapeutic health behaviors to manage the physical and emotional challenges posed by chronic diseases in daily life (5). Given the long-term and often incurable nature of chronic diseases, patients must engage in preventive interventions and health management activities within family and community settings during stable periods. Effective management of chronic diseases is essential to prevent deterioration and complications, making it a key public health priority. However, China continues to face significant challenges in chronic disease prevention and management (6, 7).

Amid rapid digitalization and technological advancements, digital health governance is gaining global momentum (8), and China's healthcare system is transitioning toward digital health (9). As of December 2023, 54.6% of older adult internet users in China had basic digital skills, and the number of internet healthcare users reached 414 million (10). The internet and social media are becoming important platforms for disseminating health information, while digital health services are increasingly integrated into daily life (11). These services enhance access to health resources for older adult patients with chronic diseases, promote healthy lifestyle habits, and improve health outcomes. Studies indicate that digital health interventions effectively enhance self-management capabilities among patients with chronic diseases (12, 13), improving health behaviors and quality of life (14, 15). The advancement of digital technologies presents new opportunities to address aging-related challenges but also places higher demands on older adult patients to achieve effective self-management in the digital era (16). Digital health governance is a key tool for improving chronic disease self-management. However, its effectiveness relies not only on technology but also on individual abilities, family support, and social resources. These factors align with the multi-level interactions outlined in the Social-Ecological Systems Theory (SET). The Social-Ecological Systems Theory (SET) suggests that (17, 18) individual capacity development is shaped by interactions within multi-level environmental systems, including the microsystem (e.g., physiological, psychological, and behavioral factors), mesosystem (e.g., family and work groups), and macrosystem (e.g., communities and organizations). Guided by SET, this study examines how demographic characteristics, disease features, social support, chronic disease management self-efficacy, and digital health literacy collectively influence self-management behaviors in older adult patients with chronic diseases.

In China, most studies employ a “variable-centered” approach, assuming sample homogeneity and focusing on group characteristics. However, this approach overlooks individual differences and group heterogeneity. Latent Profile Analysis (LPA), a “person-centered” method, identifies group heterogeneity by analyzing shared response patterns and classifying individuals into distinct subgroups. This approach improves classification accuracy and captures subgroup characteristics (19, 20). Therefore, this study uses LPA to explore the latent profile characteristics of self-management behaviors in older adult patients with chronic diseases. It further analyzes the influencing factors of each subgroup based on SET to provide evidence for developing targeted intervention strategies.

## 2 Participants and methods

### 2.1 Participants

This study employed convenience sampling from October 2023 to May 2024. Older adult patients with chronic diseases were recruited from three tertiary hospitals in Anhui Province. The inclusion criteria were: (1) age ≥60 years; (2) a confirmed chronic disease diagnosis based on ICD-10 criteria; including but not limited to hypertension, diabetes mellitus, coronary artery disease, cancer, chronic obstructive pulmonary disease, and osteoarthritis; (3) the ability to use smart devices (e.g., mobile internet); (4) clear consciousness and normal communication or reading abilities; (5) informed consent and willingness to participate. Exclusion criteria included: (1) severe cognitive, linguistic, auditory, or mental impairments; (2) being in an acute phase of a major illness or critical condition with severe complications; (3) extended or difficult communication issues; (4) concurrent participation in other studies. Based on Kendall's multi-factorial research sample size method (21), the required sample size ranged from 300 to 450 participants, accounting for a 20% dropout rate. The study included 25 independent variables. The Ethics Committee of Bengbu Medical University approved the study ([2023] No. 369), and all participants provided informed consent.

### 2.2 Research tools

Research tools were developed from literature reviews and group discussions, incorporating factors influencing the self-management behaviors of older adult patients with chronic diseases, in line with social-ecological system theory (microsystem, mesosystem, and macrosystem).

#### 2.2.1 General information questionnaire

A team-designed questionnaire collected general demographic information (age, gender, education, marital status, etc.), disease characteristics (number of chronic diseases, years of illness, etc.), and usage of digital devices and the internet (number of devices, daily internet use, perception of digital health information, etc.).

#### 2.2.2 Chronic disease self-management study measures (CDSM)

Developed by Lorig et al. (22), this scale assesses three dimensions of self-management: exercise (six items), cognitive symptom management (six items), and communication with doctors (three items), with a total of 15 items. The exercise dimension uses a Likert five-point scale, while the other two dimensions use a six-point scale. Higher scores indicate better self-management behavior. The Cronbach's alpha coefficient of the scale was 0.762, and in this study, it was 0.819.

#### 2.2.3 Digital health literacy assessment scale (DHL)

Developed by Siqi (23), this scale measures digital health literacy in China's older adult. It consists of 15 items across three dimensions: access and evaluation of digital health information (nine items), interactive ability (three items), and application ability (three items), using a Likert 5-point scale. Scores range from 15 to 75, with higher scores indicating higher DHL. The Cronbach's alpha for the scale was 0.941, and in this study, it was 0.906.

#### 2.2.4 Social support rating scale (SSRS)

Developed by Shuiyuan (24), this scale measures social support across three dimensions: objective support (three items), subjective support (four items), and the utilization of support (three items). The total score ranges from 12 to 83, with higher scores indicating higher social support levels. The Cronbach's alpha coefficient of the scale was 0.896 (25), and in this study, it was 0.720.

#### 2.2.5 Self-efficacy for managing chronic disease 6-item scale (SEMCD-6)

Developed by Lorig et al. (26), this scale assesses self-efficacy for chronic disease management. It includes six items across two dimensions: self-efficacy in symptom management (four items) and general disease management (two items). Each item is rated on a 1–10 scale, with higher scores indicating greater self-efficacy. The Cronbach's alpha for the scale was 0.91, and in this study, it was 0.919.

#### 2.2.6 Psychological distress scale (the 10-item Kessler Psychological Distress Scale, Kessler10)

The Kessler Psychological Distress Scale (K10) measures mental health over the past month (27). The Chinese version, validated by Zhou et al. (28), has been used for older adult populations in China. Scores range from 10 to 50, with higher scores indicating greater distress. The Cronbach's alpha for the scale was 0.8011 (29), and in this study, it was 0.900.

#### 2.2.7 Age-adjusted Charlson Comorbidity Index (aCCI)

This index quantifies comorbidities based on the number and severity of diseases. Scores are assigned based on disease type (1, 2, 3, or 6 points) and age group (<50, 50–<60, 60–<70, 70–<80, 80–<90, ≥90 years). Higher scores indicate a greater comorbidity burden (30, 31). Information was gathered from electronic medical records and physician feedback.

### 2.3 Data collection and quality control

After obtaining approval from department heads, researchers used a standardized script to explain the study to participants, emphasizing their right to withdraw at any time without consequences. Questionnaires were distributed to patients who consented, and they completed them independently or with assistance if required. Disease-related information was supplemented using electronic medical records. All questionnaires were reviewed for completeness, and any missing data were clarified. Questionnaires were deemed invalid if more than 10% of items were incomplete, extreme values were consistently selected, or multiple-choice items had multiple answers (32, 33). To protect participant confidentiality, personal identifying information, such as names and contact details, was removed. The data were securely stored and accessed only by authorized researchers through password-protected electronic systems.

### 2.4 Statistical methods

Data were verified and entered by two researchers. Latent profile modeling was conducted using Mplus 8.3 software, and chi-square tests, one-way ANOVA, and multivariate logistic regression were performed using SPSS 26.0. Latent profile modeling began with a baseline model (one category), gradually increasing the number of categories and testing model fit indices for each. The optimal model was selected based on fit indices, previous findings, and clinical significance. Criteria included: (1) Akaike Information Criterion (AIC), Bayesian Information Criterion (BIC), and adjusted BIC (aBIC), where lower values indicate better fit; (2) entropy, where values closer to 1 indicate better classification accuracy; (3) Lo-Mendell-Rubin Adjusted Likelihood Ratio Test (LMR) and Bootstrap Likelihood Ratio Test (BLRT), with p < 0.05 indicating a K-class model is better than a K-1 class model. Data cleaning and analysis were conducted with SPSS 26.0. In this study, skewness and kurtosis were used to test univariate normality, while the Mardia test assessed multivariate normality. Normally distributed data were presented as mean ± standard deviation and one-way ANOVA was used for group comparisons. Categorical data were expressed as frequencies or percentages, and chi-square tests or Fisher's exact test were used for group comparisons. Multivariate logistic regression explored factors influencing CDSM, with *p* < 0.05 considered significant.

## 3 Results

### 3.1 Normality test

Univariate normality was evaluated using skewness and kurtosis. The results showed that all variables had kurtosis absolute values below 10 and skewness absolute values below 3, satisfying Kline's criteria and indicating approximate normality (34). Multivariate normality was assessed using the Mardia test. The standardized multivariate kurtosis coefficient (std-MK) was 4.6928, which satisfies Byrne's (35) criterion of std-MK < 5 for multivariate normality (see Table 1).

**Table 1:** Statistics of normality.

**Variables**	**Skewness**	**Skewness SE**	**Kurtosis**	**Kurtosis SE**
DHL	−0.021	0.106	−0.752	0.211
SSRS	0.338	0.106	−0.184	0.211
SEMCD-6	−0.264	0.106	−0.508	0.211
K10	1.039	0.106	1.486	0.211
aCCI	0.625	0.106	−0.141	0.211
CDSM	0.426	0.106	0.113	0.211

### 3.2 Common method bias test

Since self-reported data was used in this study, common method bias may occur. To mitigate this, participants were informed about anonymity and confidentiality before the survey. Questionnaire items were balanced in sequence, and disease-related data were gathered from multiple sources, including electronic medical records. The Harman single-factor test revealed 13 factors with eigenvalues >1, with the first factor explaining 26.833% of the variance, below the recommended 40% threshold. Thus, no severe common method bias was detected.

### 3.3 General information of study participants

Of the 550 questionnaires distributed, 14 were excluded as invalid, leaving 536 valid responses, with a response rate of 97.45%. Table 2 shows the general characteristics of the 536 older adult patients with chronic diseases.

**Table 2:** Univariate analysis of general characteristic and latent categories of self-management behaviors in older adult patients with chronic diseases.

**Variables**	**Number (%)**	**C1 (*n* = 269)**	**C2 (*n* = 56)**	**C3 (*n* = 211)**	**χ^2^/*F***	***p***
**Gender**						
Male	304 (56.7)	148 (55.0)	31 (55.4)	125 (59.2)	0.906	0.636
Female	232 (43.3)	121 (45.0)	25 (44.6)	86 (40.8)		
**Age**						
60–64	238 (44.4)	110 (40.9)	25 (44.6)	103 (48.8)	15.629^#^	0.040
65–69	101 (18.8)	50 (18.6)	6 (10.7)	45 (21.3)		
70–74	107 (20.0)	53 (19.7)	14 (25.0)	40 (19.0)		
75–79	59 (11.0)	33 (12.3)	9 (16.1)	17 (8.1)		
≥80	31 (5.8)	23 (12.3)	2 (16.1)	6 (8.1)		
**Place of residence**						
Rural	86 (16.0)	49 (18.2)	17 (30.4)	20 (9.5)	25.982	<0.001
Town	128 (23.9)	70 (26.0)	17 (30.4)	41 (19.4)		
City	322 (60.1)	150 (55.8)	22 (39.3)	150 (71.1)		
**Marital status**						
Married	445 (83.0)	214 (79.6)	50 (89.3)	181 (85.8)	4.995	0.082
Divorced/widowed/other	91 (17.0)	55 (20.4)	6 (10.7)	30 (14.2)		
**Multiple-child**						
No	233 (43.5)	95 (35.3)	18 (32.1)	120 (56.9)	25.625	<0.001
Yes	303 (56.5)	174 (64.7)	38 (67.9)	91 (43.1)		
**Education level**						
Primary school or below	207 (38.6)	133 (49.4)	22 (39.3)	52 (24.6)	49.983	<0.001
Middle school	178 (33.2)	85 (31.6)	18 (32.1)	75 (35.5)		
High/vocational school	97 (18.1)	40 (14.9)	13 (23.2)	44 (20.9)		
College or above	54 (10.1)	11 (4.1)	3 (5.4)	40 (19.0)		
**Occupation before retirement**						
Farmer	162 (30.2)	105 (39.0)	22 (39.3)	35 (16.6)	64.260^#^	<0.001
Worker	74 (13.8)	42 (39.0)	6 (39.3)	26 (16.6)		
State-owned enterprise/institution/civil servant	167 (31.2)	59 (21.9)	8 (14.3)	100 (47.4)		
Private enterprise employee	52 (9.7)	24 (21.9)	13 (14.3)	15 (47.4)		
Private enterprise employee	71 (13.2)	34 (21.9)	6 (14.3)	31 (47.4)		
Other	10 (1.9)	5 (1.9)	1 (1.8)	4 (1.9)		
**Medical insurance type**						
Employee insurance	292 (54.5)	124 (46.1)	32 (57.1)	136 (64.5)	31.705^#^	<0.001
Resident insurance	231 (43.1)	143 (53.2)	24 (42.9)	64 (30.3)		
Commercial insurance or other	13 (2.4)	2 (0.7)	0 (0.0)	11 (5.2)		
**Average monthly income (RMB)**						
<1,000	130 (24.3)	93 (34.6)	16 (28.6)	21 (10.0)	67.635	<0.001
1,000–2,999	101 (18.8)	54 (20.1)	18 (32.1)	29 (13.7)		
3,000–4,999	135 (25.2)	62 (23.0)	12 (21.4)	61 (28.9)		
≥5,000	170 (31.7)	60 (22.3)	10 (17.9)	100 (47.4)		
**Smoking**						
No (never/quit)	426 (79.5)	209 (77.7)	49 (87.5)	168 (79.6)	2.736	0.255
Yes	110 (20.5)	60 (22.3)	7 (12.5)	43 (20.4)		
**Drinking**						
No (never/quit)	110 (20.5)	208 (77.3)	42 (75.0)	164 (77.7)	0.189	0.910
Yes	122 (22.8)	61 (22.7)	14 (25.0)	47 (22.3)		
**Number of chronic diseases**						
One	148 (27.6)	74 (27.5)	14 (25.0)	60 (28.4)	0.264	0.876
Two or more	388 (72.4)	195 (72.5)	42 (75.0)	151 (71.6)		
**Duration of illness (years)**						
<1	85 (15.9)	40 (14.9)	8 (14.3)	37 (17.5)	8.777	0.361
1–3	104 (19.4)	55 (20.4)	14 (25.0)	35 (16.6)		
3–5	59 (11.0)	26 (9.7)	11 (19.6)	22 (10.4)		
5–10	62 (11.6)	31 (11.5)	5 (8.9)	26 (12.3)		
>10	226 (42.2)	117 (43.5)	18 (32.1)	91 (43.1)		
**Type of daily medication (type)**						
0	126 (23.5)	70 (26.0)	10 (17.9)	46 (21.8)	16.126^#^	0.018
1–3	320 (59.7)	143 (53.2)	43 (76.8)	134 (63.5)		
4–6	66 (12.3)	42 (15.6)	1 (1.8)	23 (10.9)		
>6	24 (4.5)	14 (5.2)	2 (3.6)	8 (3.8)		
**Self-rated health status**						
Good/very good	243 (45.3)	108 (40.1)	30 (53.6)	105 (49.8)	19.485	0.001
Average	196 (36.6)	93 (34.6)	20 (35.7)	83 (39.3)		
Bad/very bad	97 (18.1)	68 (25.3)	6 (10.7)	23 (10.9)		
**Attended chronic disease health lectures**						
No	246 (45.9)	143 (53.2)	19 (33.9)	84 (39.8)	12.093	0.002
Yes	290 (54.1)	126 (46.8)	37 (66.1)	127 (60.2)		
**Number of digital devices used**						
In one	138 (25.7)	73 (27.1)	26 (46.4)	39 (18.5)	18.624	<0.001
Two or more	398 (74.3)	196 (72.9)	30 (53.6)	172 (81.5)		
**Average daily online time (hours)**						
<1	137 (25.6)	92 (34.2)	12 (21.4)	33 (15.6)	36.999	<0.001
1–2	154 (28.7)	80 (29.7)	16 (28.6)	58 (27.5)		
2–3	104 (19.4)	51 (19.0)	9 (16.1)	44 (20.9)		
3–4	85 (15.9)	31 (11.5)	9 (16.1)	45 (21.3)		
>4	56 (10.4)	15 (5.6)	10 (17.9)	31 (14.7)		
**Perception of digital health information**						
**Usefulness**						
Not useful/somewhat useful	101 (18.8)	64 (23.8)	16 (28.6)	21 (10.0)	30.668	<0.001
Average	157 (29.3)	57 (21.2)	16 (28.6)	84 (39.8)		
Quite/very useful	278 (51.9)	148 (55.0)	24 (42.9)	106 (50.2)		
**Ease of use**						
Very/quite difficult	207 (38.6)	145 (53.9)	18 (32.1)	44 (20.9)	58.011	<0.001
Average	183 (34.1)	73 (27.1)	17 (30.4)	93 (44.1)		
Quite/very easy	146 (27.2)	51 (19.0)	21 (37.5)	74 (35.1)		

# Fisher exact probability method.

### 3.4 Latent profile analysis of older adult patients with chronic diseases

This study used 15 items from the Self-Management Behavior Scale for chronic disease patients as manifest indicators. Starting with the baseline model (1 category), 1 to 5 latent class models were sequentially established (see Table 3). As the categories increased, AIC, BIC, and aBIC values for the five models decreased, while entropy remained above 0.9. The LMR test value of model 4 was not statistically significant (*p* > 0.05), indicating that the 4-category model was not better than the 3-category model. In model 3, both the LMR and BLRT were significant (*p* < 0.05), and the sample sizes of patients in each category were 269, 56, and 211, respectively, accounting for more than 5% of the total sample (19). Considering the fit indices, inflection points, and clinical significance, model 3 was chosen as the optimal model.

**Table 3:** Latent profile analysis models and fit indices.

**Model**	**AIC**	**BIC**	**aBIC**	**Entropy**	**LMR (p)**	**BLRT (p)**	**Proportion**
1	24,537.554	24,666.078	24,570.848	–	–	–	1.000
2	22,994.298	23,191.368	23,045.349	1	<0.001	<0.001	0.914/0.086
3	21,752.887	22,018.503	21,821.695	0.949	<0.001	<0.001	0.502/0.086/0.412
4	21,253.742	21,587.904	21,340.306	0.946	0.0859	<0.001	0.351/0.0914/0.472/0.086
5	20,846.387	21,249.096	20,950.709	0.950	0.0146	<0.001	0.198/0.015/0.379/0.323/0.086

AIC, Akaike's information criterion; BIC, Bayesian information criterion; aBIC, adjusted Bayesian information criterion; LMR, Lo–Mendell–Rubin likelihood ratio; BLRT, bootstrap likelihood ratio test.

**Figure 1:**
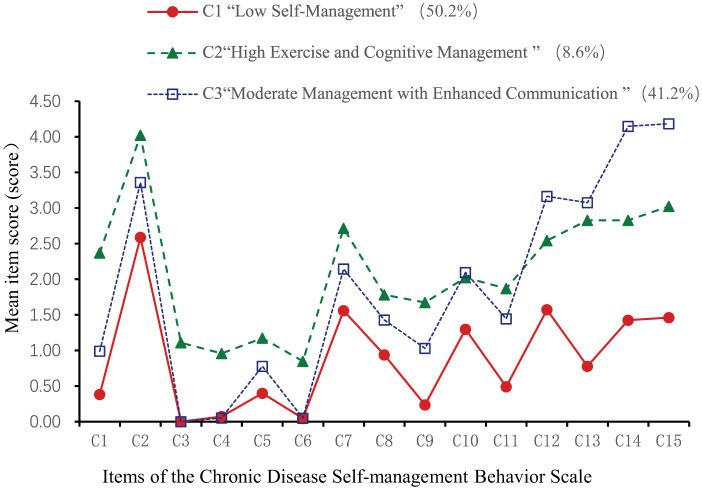
Latent profile chart of self-management behaviors in older adult patients with chronic diseases.

Based on the three latent profiles of self-management behaviors in older adult patients with chronic diseases, a latent profile plot was drawn (see Figure 1), and categories were named according to their characteristics. As shown in Figure 1, the score patterns of patients in the three latent classes on items 1–11 are similar. There were 268 patients in category C1 (50.2%). These patients scored low across all items, suggesting a simple exercise routine focused on low-intensity activities like walking, with minimal aerobic training. In cognitive symptom management, they tended to ignore symptoms and adopt passive coping strategies, with minimal communication with doctors. Therefore, this group was named “Low Self-Management Behavior Group” There were 56 patients in category C2 (8.6%), who scored highest on items 1–11 but slightly lower on item 12 (“talking to oneself positively”). This indicated strong exercise and cognitive symptom management but weaker psychological self-management and moderate communication with doctors. This group was named “High Exercise and Cognitive Management Group” There were 211 patients in category C3 (41.2%), who scored moderately on items 1–11 but highest on items 12–15, showing that they valued self-perception and proactively communicated with doctors. This group was named “Moderate Management with Enhanced Communication Group.”

### 3.5 Univariate analysis of the latent profiles of self-management behaviors in older adult patients with chronic diseases

Statistically significant differences (*p* < 0.05) were found in age, place of residence, number of children, education level, pre-retirement occupation, type of medical insurance, monthly income, number of daily medications, self-assessed health status, participation in chronic disease health seminars, number of digital devices used, average daily internet usage time, and perceived digital health information among different patient categories (see Table 2). Significant differences (*p* < 0.05) were also observed in digital health literacy, social support (SSRS), chronic disease management self-efficacy (SEMCD-6), psychological distress (K10), and self-management behavior (CDSM) across the three latent categories (see Table 4).

**Table 4:** Comparison of DHL, SSRS, SEMCD-6, K10, aCCI and CDSM scores among different latent categories in older adult patients with chronic diseases (*n* = 536, X ± *S*).

**Variables**	**X ± *S***	**Latent profile**			***F***	***p***
		**C1 (*****n*** = **269)**	**C2 (*****n*** = **56)**	**C3 (*****n*** = **211)**		
DHL	41.36 ± 12.8	34.99 ± 11.21	46.48 ± 12.56	48.14 ± 10.51	89.862	<0.001
SSRS	29.67 ± 6.3	27.82 ± 5.42	28.32 ± 7.6	32.38 ± 6.02	36.684	<0.001
SEMCD-6	6.53 ± 2.07	5.85 ± 2.03	6.08 ± 2.04	7.51 ± 1.73	46.503	<0.001
K10	21.59 ± 7.16	23.07 ± 7.35	19.36 ± 5.93	20.29 ± 6.82	12.502	<0.001
aCCI	3.4 ± 1.23	3.41 ± 1.24	3.46 ± 1.13	3.37 ± 1.23	0.155	0.856
CDSM	20.87 ± 10.37	13.07 ± 5.93	34.71 ± 7.74	27.15 ± 6.7	426.889	<0.001

### 3.6 Multivariate analysis of the latent profiles of self-management behaviors in older adult patients with chronic diseases

Using self-management behavior in older adult patients with chronic diseases as the dependent variable, digital health literacy, SSRS, K10, SEMCD-6, and aCCI were included as continuous variables. Additionally, demographic information, disease characteristics, and variables with statistically significant results from univariate analyses related to internet and digital device usage were incorporated into the multivariate logistic regression model. The coding of independent variables is detailed in Table 5.

**Table 5:** Assignment of independent variables in multiple logistic regression.

**Variables**	**Assignment of variables**
Latent categories	C1 = 1, C2 = 2, C3 = 3
Place of residence	Rural = 1, town = 2, city = 3
Education level	Primary school or below = 1, middle school = 2, high/vocational school = 3, college or above = 4
Self-rated health status	Good/very good = 1, average = 2, bad/very bad = 3 Average bad/very bad = 1, average = 2, poor/very bad = 3
Number of digital devices used	One = 1, two or more = 2
Average daily online time (hours)	<1 = 1, 1–2 = 2, 2–3 = 3, 3–4 = 4, >4 = 5
Perception of digital health information (usefulness)	Not useful/somewhat useful = 1, average = 2, quite/very useful = 3

The results of the multivariate logistic regression analysis are summarized in Table 6, indicating that multiple factors influenced the self-management behaviors of older adult patients with chronic diseases across different categories. Compared to C1 and C2, patients with higher SSRS and SEMCD-6 scores were likelier to belong to the C3 category. In comparison to C1 and C3, those living in rural areas (OR = 6.456, 95% CI: 2.322–17.947) or towns (OR = 2.655, 95% CI: 1.101–6.402), those using only one type of digital device (OR = 3.555, 95% CI: 1.511–8.364), those perceiving digital health information as “useless/not very useful” (OR = 3.524, 95% CI: 1.369–9.069), and those with higher digital health literacy scores (OR = 1.176, 95% CI: 1.128–1.226) were more likely to belong to the C2 category. Compared to C1, individuals with higher aCCI scores (OR = 1.560, 95% CI: 1.127–2.161) were more likely to belong to the C2 category, while those experiencing greater psychological distress (K10 scores) were more likely to belong to the C1 category (OR = 0.930, 95% CI: 0.874–0.990). Compared to C1, patients using only one type of digital device (OR = 1.877, 95% CI: 1.036–3.404), those perceiving digital health information as “moderately useful” (OR = 1.884, 95% CI: 1.12–3.17), those with higher digital health literacy scores (OR = 1.112, 95% CI: 1.081–1.143), and those with higher aCCI scores (OR = 1.360, 95% CI: 1.103–1.677) were more likely to belong to the C3 category. When comparing C2 and C3, individuals living in rural areas (OR = 6.470, 95% CI: 2.257–18.547) or towns (OR = 2.605, 95% CI: 1.16–6.169), those perceiving digital health information as “useless/not very useful” (OR = 2.674, 95% CI: 1.006–7.108), and those self-assessing their health status as “good/very good” (OR = 3.835, 95% CI: 1.016–14.469) were more likely to belong to the C2 category. Conversely, individuals with higher K10 scores were more likely to belong to the C3 category (OR = 0.940, 95% CI: 0.883–0.999).

**Table 6:** Multiple logistic regression analysis of latent categories.

	**β**	**SE**	**Wald χ^2^**	***p***	**OR**	**95% CI**
**C2 vs. C1** ^*^						
Intercept	−9.163	2.085	19.316	0.000		
Rural	1.865	0.522	12.782	0.000	6.456	2.322–17.947
Town	0.977	0.449	4.732	0.030	2.655	1.101–6.402
Use only one number of digital devices	1.268	0.437	8.44	0.004	3.555	1.511–8.364
Usefulness (not useful/somewhat useful)	1.26	0.482	6.82	0.009	3.524	1.369–9.069
DHL	0.162	0.021	58.304	0.000	1.176	1.128–1.226
aCCI	0.445	0.166	7.179	0.007	1.560	1.127–2.161
K10	−0.072	0.032	5.172	0.023	0.930	0.874–0.99
**C3 vs. C1** ^*^						
Intercept	−10.219	1.382	54.696	0.000		
Use only one number of digital devices	0.63	0.304	4.306	0.038	1.877	1.036–3.404
Usefulness (average)	0.633	0.266	5.692	0.017	1.884	1.12–3.17
DHL	0.106	0.014	56.88	0.000	1.112	1.081–1.143
SSRS	0.065	0.022	8.715	0.003	1.067	1.022–1.114
SEMCD-6	0.329	0.08	17.049	0.000	1.390	1.189–1.625
aCCI	0.308	0.107	8.282	0.004	1.360	1.103–1.677
**C2 vs. C3** ^*^						
Intercept	1.056	2.043	0.267	0.605		
Rural	1.867	0.537	12.077	0.001	6.470	2.257–18.547
Town	0.957	0.44	4.737	0.030	2.605	1.1–6.169
Usefulness (not useful/somewhat useful)	0.984	0.499	3.888	0.049	2.674	1.006–7.108
Self-rated health status (good/very good)	1.344	0.677	3.936	0.047	3.835	1.016–14.469
DHL	0.056	0.02	8.107	0.004	1.058	1.018–1.1
SSRS	−0.061	0.031	3.998	0.046	0.940	0.886–0.999
SEMCD-6	−0.551	0.119	21.309	0.000	0.576	0.456–0.728
K10	−0.062	0.031	3.918	0.048	0.940	0.883–0.999

* For the reference group; the highest assignment was used as the control for all independent variables.

## 4 Discussion

### 4.1 Current status of self-management behaviors in older adult patients with chronic diseases

#### 4.1.1 Overall level of self-management behaviors

The self-management behavior score of older adult patients with chronic diseases in this study was (20.87 ± 10.37), reflecting a generally low level, lower than those reported in previous studies (36–38). This discrepancy may stem from the characteristics of the study population. Participants were recruited from three hospitals in Anhui Province, with 72.4% (388 cases) diagnosed with two or more chronic diseases and an aCCI score of (3.4 ± 1.23), indicating severe multimorbidity. Compared to patients from economically developed regions such as Beijing or Shanghai in earlier studies (36–38), the older adult patients in this study exhibited lower self-management awareness and abilities.

Research suggests that multimorbidity can lead to physical function decline and increased physiological burden due to long-term disease interactions (39).

Additionally, these patients often face reduced psychological resilience, impaired social functioning, and limited access to resources, making them more susceptible to negative emotions like anxiety and depression, which further undermine their health and daily management capabilities (40). In this study, 76.5% of patients (*n* = 410) required at least one daily medication, adding complexity to their routine management. Negative emotions have been shown to reduce medication adherence, heightening health risks, including increased readmission rates and prolonged hospital stays (41), ultimately impairing self-management and quality of life (42).

In conclusion, older adult patients with chronic diseases in this study demonstrated low self-management behavior levels. Healthcare professionals should enhance health education, address patients' physical and mental health needs, and offer targeted psychological support and health education tailored to clinical settings. Promoting patient engagement and initiative may help improve self-management capabilities.

#### 4.1.2 Self-management behaviors can be divided into three potential profiles

This study classified the self-management behaviors of older adult patients with chronic diseases into three categories: C1, “Low Self-Management Behavior Group”; C2, “High Exercise and Cognitive Management Group”; and C3, “Moderate Management and Enhanced Communication Group.” This classification indicates significant heterogeneity in self-management behaviors among these patients.

The C1 group comprised 50.2% of the participants, displaying generally low self-management levels, reflecting a lack of proactive health awareness, poor disease management cognition, and insufficient communication with healthcare providers. Higher K10 scores in C1 patients indicate greater psychological distress, which likely impairs their self-management behaviors. This aligns with previous studies showing that negative emotions hinder self-management in chronic disease patients (40, 43). The C2 group represented 8.6% and demonstrated relatively high self-management levels, with profile analysis showing the highest scores for items 1–11, indicating better performance in exercise and cognitive symptom management. However, the score for item 12, “Positively talk to yourself,” was lower than in the C3 group, suggesting that psychological self-management requires further strengthening in this group. Consequently, healthcare providers should guide these patients to focus on their internal feelings, fostering a positive and self-compassionate attitude to manage symptoms, emotions, and behaviors. Items 13–15 received lower scores than the C3 group, indicating that this group communicates less with healthcare providers and has a lower frequency of obtaining health information. The C3 group accounted for 41.2% of the participants, displaying moderate self-management levels. Profile analysis revealed that items 12–15 scored the highest, indicating this group has higher digital health literacy. These patients tend to adopt an active approach to managing negative emotions, engage in communication with healthcare providers, and proactively seek health-related information, thereby promoting self-management. Our findings are consistent with insights provided by previous studies (44) that highlight the critical role of self-management behaviors in improving health outcomes. Similar to the study's focus on home-based cardiac rehabilitation, our research highlights the necessity of tailored interventions for older adult patients with diverse chronic conditions. Moreover, the study underscores the value of validated tools for assessing self-management behaviors, which is essential for designing effective interventions. Therefore, healthcare providers should develop personalized intervention plans, offer education and training on disease management, enhance communication skills for “communication-deficient” patients, and increase self-management awareness for “passive dependent” patients.

### 4.2 Influencing factors of latent profiles of self-management behaviors

Based on social-ecological systems theory, this study explored multiple influencing factors of self-management behaviors in older adult patients with chronic diseases, categorized into microsystem (individual level), mesosystem (social support), and macrosystem (living environment).

#### 4.2.1 Microsystem factors

The microsystem encompasses individual-level factors, primarily physiological, psychological, and internet usage.

(1) Physiological factors

The findings indicate that, compared to the “Low Self-Management Behavior Group,” higher aCCI scores correlate with a greater likelihood of belonging to either the “High Exercise and Cognitive Management Group” or the “Moderate Management and Enhanced Communication Group.” As the number of chronic conditions increases, older adult patients with multimorbidity often experience a gradual decline in physical and cognitive functions (45), making them more vulnerable to the challenges posed by their conditions. This exacerbates their disease burden and increases the need for health information, thereby complicating self-management (41). To reduce their disease burden, older adult patients with multimorbidity typically engage actively with healthcare professionals to obtain health-related knowledge, improve their understanding of their conditions, and adopt more proactive self-management strategies. These strategies may include regular exercise to maintain or improve health, as well as behavioral and mental adjustments to meet the demands of chronic disease management. In the “moderate management and communication enhancement group,” older adult patients demonstrate an intermediate level of self-management, reflecting an overall proactive attitude. They actively seek disease-related information from healthcare providers and communicate promptly and effectively. Therefore, healthcare professionals should offer positive reinforcement and encouragement to further enhance their self-management skills. In the “high physical activity and cognitive management group,” older adult patients exhibit a high level of self-management overall, but communication with doctors remains an area for improvement. These patients likely possess a solid foundation of disease-related knowledge, and when their condition is stable, they may be less sensitive to the need for additional disease-related information. Healthcare providers should therefore intensify health education efforts, monitor their physical and mental wellbeing, and encourage active communication.

(2) Psychological factors

This study found that higher K10 scores, indicating more severe psychological distress, were associated with a greater likelihood of belonging to either the “Low Self-Management Behavior Group” or the “Moderate Management and Enhanced Communication Group.” Psychological distress, including anxiety and depression, can negatively affect patients' cognitive function and emotional states, possibly suppressing their self-management motivation and self-efficacy, consistent with previous research (39, 42). Patients in the “low self-management behavior group” may become more passive due to heightened psychological distress, lacking both the awareness and ability to actively manage their health and chronic diseases (41). The results indicate that patients in the “moderate management and communication enhancement group” possess moderate self-management abilities and relatively high social support. When facing greater psychological distress, these patients tend to rely on their support networks, seeking health information and emotional support through communication with healthcare providers, which helps alleviate anxiety and unease (36).

The study further reveals that, compared to the “low self-management behavior group” and the “high physical activity and cognitive management group,” patients with higher SEMCD-6 scores are more likely to belong to the “moderate management and communication enhancement group.” These patients actively engage with healthcare providers, not only obtaining professional information but also clarifying their health information needs, thus reducing the difficulty of acquiring necessary information and enhancing self-efficacy. Additionally, effective communication fosters trust in the doctor-patient relationship, encouraging ongoing health information seeking and improving adherence to medical advice. This, in turn, boosts the effectiveness and confidence in managing chronic diseases. These findings align with prior research, suggesting that self-efficacy in chronic disease management is a protective factor for self-management behaviors (46).

(3) Digital skills

Patients who use fewer digital devices, rate digital health information as “not useful” or “not very useful,” and possess higher digital health literacy scores are more likely to belong to the “High Exercise and Cognitive Management Group.” With the rapid growth of digital media and the widespread adoption of smartphones, the internet has become a key platform for disseminating health information (11), broadening the channels through which health information is accessed. Most older adult patients now possess basic digital skills (10). In this study, participants were drawn from hospitals implementing digital healthcare services. During their visits, older adult patients, guided by volunteers and healthcare providers, learned to use online platforms for scheduling appointments and making payments, thereby improving their digital health literacy. This, in turn, enhanced their understanding of their health conditions and facilitated self-management. These findings are consistent with previous research, which suggests that digital health literacy (DHL) fosters healthier lifestyles and improves quality of life (23).

However, the digital health field in China remains in its infancy, lacking standardized regulations, which results in inconsistent information quality. Moreover, frequent online fraud incidents and other challenges have contributed to low levels of trust and perceived usefulness of digital health information among older adult patients, leading to anxiety and other negative emotions associated with digital technology use. These issues align with prior studies, which highlight how information overload, content homogeneity, and technology anxiety hinder the acceptance and effective use of digital health information (11, 47). Thus, healthcare providers should offer trustworthy, accessible, and relevant information tailored to the digital health literacy levels of older adult patients, thereby fostering greater engagement in health information acquisition.

#### 4.2.2 Mesosystem factors

The mesosystem includes small-scale groups that impact individuals. This study mainly analyzed the effect of social support on the self-management behaviors of older adult patients with chronic diseases. This study found that patients with higher social support scores were more likely to belong to the “Moderate Management and Enhanced Communication Group” than either the “Low Self-Management Behavior Group” or the “High Exercise and Cognitive Management Group.” These patients demonstrate moderate self-management skills and communicate effectively with doctors, gaining professional medical information that alleviates the difficulty of acquiring necessary information. Additionally, they benefit from strong social support, including emotional, informational, and material resources, which promotes more proactive and effective chronic disease self-management. This finding aligns with previous research indicating that peer support and social networks are key factors in enhancing self-management among older adult patients with chronic diseases (48). Therefore, healthcare providers should offer positive reinforcement and acknowledgment to sustain patients' motivation for self-management and further improve their capabilities.

#### 4.2.3 Macrosystem factors

The macrosystem encompasses broader environmental factors, including community and organizational aspects. The study results indicate that patients who have lived in rural or town areas for extended periods are more likely to belong to the “High Exercise and Cognitive Management Group.” Patients in this group show higher levels of physical activity and cognitive symptom management, particularly excelling in endurance activities like walking. In contrast to previous findings suggesting that rural older adult patients with chronic diseases in Beijing engage in limited physical activity and have poor cognitive symptom management, this study presents a different perspective (37). This variation may stem from regional disparities in China's development, which influence the lifestyles and work patterns of older adult patients. The participants in this study, primarily manual laborers from economically underdeveloped rural or town areas, often engage in agricultural or livestock-related work, contributing to their higher levels of physical activity, especially walking. Remote areas, however, face shortages in medical resources, with healthcare personnel both limited in number and unevenly distributed (2). This lack of resources results in fewer chronic disease health education programs, restricting patients' access to timely and effective medical services and limiting opportunities for doctor-patient communication. Consequently, these patients often take proactive steps to improve their understanding of their conditions and rely on physical activity to maintain health and support self-management. Under China's current healthcare system, “long waiting times and short consultation times” are common. Older patients with lower education levels and limited health literacy often struggle to fully understand medical information during brief consultations. Furthermore, differing perspectives between doctors and patients exacerbate communication challenges. These barriers can prevent patients from clearly expressing their conditions or comprehending medical advice, reducing the quality of interactions and hindering their motivation for self-management. Nonetheless, national policies aimed at improving healthcare equity, particularly by enhancing services in rural and remote areas (6), may gradually address these issues in the future.

## 5 Limitations

This study has several limitations. First, as a cross-sectional study, it captures only a snapshot of self-management behaviors in older adult patients with chronic diseases, without reflecting their dynamic changes over time. Second, the sample was restricted to older adult patients from three hospitals in Anhui Province, potentially limiting the external validity of the results. Furthermore, 44.4% of participants (*n* = 238) were younger older adult individuals (aged 60–64 years), and the “High Exercise and Cognitive Management Group” included a small sample size (*n* = 56), which may introduce bias. To address these issues, future research should aim for a more balanced sample distribution or focus on specific age groups (e.g., 60–70 years) to better understand the characteristics and needs of these populations. Future studies should adopt multi-center, large-sample longitudinal designs to investigate the dynamic trajectories of self-management behaviors in older adult patients with chronic diseases, thereby improving the generalizability and applicability of the findings in China.

## 6 Conclusion

This study applied latent profile analysis to classify the self-management behaviors of older adult patients with chronic diseases into three distinct types: the “Low Self-Management Behavior Group,” the “High Exercise and Cognitive Management Group,” and the “Moderate Management and Communication-Enhanced Group.” Notable heterogeneity was identified among the three groups. Healthcare professionals should tailor targeted and individualized intervention plans based on the characteristics and influencing factors of these groups to enhance patients' proactive health capabilities and self-management skills, thereby advancing the goal of healthy aging.

## Data Availability

The datasets presented in this article are not readily available because the dataset generated and analyzed during the current study contains personal health information from older adult patients with chronic diseases, and its use is subject to strict confidentiality agreements. Due to privacy and ethical concerns, the data cannot be made publicly available. Access to the dataset is restricted and available only upon reasonable request, with approval from the ethics committee of the participating institutions, in compliance with applicable data protection regulations. Requests to access the datasets should be directed to Xiu Mu Yang, 0700013@bbmu.edu.cn.
